# Supplementary material for: Fluvial transport potential of shed and root-bearing dinosaur teeth from the late Jurassic Morrison Formation
Source: PeerJ. 2014 Apr 10;2:e347. doi: 10.7717/peerj.347 (PMC3994629; doi:10.7717/peerj.347)
Supplement: Text S1 [file peerj-02-347-s001.docx]

SUPPLEMENTAL DATA

TEXT S1: 3D Scan Data

UWO-VPC-2013-01 - “Allosaur tooth – rooted” - Scanned with a Nextengine Desktop 3D Scanner and Scan Studio Pro (NextEngine) on high resolution settings. Model composed of 72,989 vertices and 145,758 faces. Saved as an *.stl file in MeshLab (v.1.3.2), converted to *pdf in Photoscan Pro. STL file available at: http://dx.doi.org/10.6084/m9.figshare.941093

UWO-VPC-2013-02 - “Allosaur tooth – shed” - Scanned with a Nextengine Desktop 3D Scanner and Scan Studio Pro (NextEngine) on high resolution settings. Model composed of 34,702 vertices and 69,344 faces. Saved as an *.stl file in MeshLab (v.1.3.2), converted to *pdf in Photoscan Pro. STL file available at: http://dx.doi.org/10.6084/m9.figshare.941092

UWO-VPC-2013-03 – “Camarasaur tooth – rooted” - Scanned with a Nextengine Desktop 3D Scanner and Scan Studio Pro (NextEngine) on high resolution settings. Model composed of 145,325 vertices and 29,102 faces. Saved as an *.stl file in MeshLab (v.1.3.2), converted to *pdf in Photoscan Pro. STL file available at: http://dx.doi.org/10.6084/m9.figshare.941095

UWO-VPC-2013-04 – “Camarasaur tooth – shed” - Scanned with a Nextengine Desktop 3D Scanner and Scan Studio Pro (NextEngine) on high resolution settings. Model composed of 69,697 vertices and 139,213 faces. Saved as an *.stl file in MeshLab (v.1.3.2), converted to *pdf in Photoscan Pro. STL file available at: http://dx.doi.org/10.6084/m9.figshare.941094
