# Supplementary material for: Fluvial transport potential of shed and root-bearing dinosaur teeth from the late Jurassic Morrison Formation
Source: PeerJ. 2014 Apr 10;2:e347. doi: 10.7717/peerj.347 (PMC3994629; doi:10.7717/peerj.347)
Supplement: Figure S2 [file peerj-02-347-s003.pdf]

Generated with Agisoft PhotoScan
